# Air Medical Transportation for Severe Acute Pancreatitis Patients over an Extra Long Distance: Is It Safe Enough?

**DOI:** 10.1155/2018/3826084

**Published:** 2018-07-02

**Authors:** Lin Gao, Jingzhu Zhang, Kun Gao, Yiyuan Pan, Xiaotao Qin, Jie Zhang, Jing Zhou, Guotao Lu, Weiqin Li, Zhihui Tong

**Affiliations:** ^1^Surgical Intensive Care Unit (SICU), Department of General Surgery, Jinling Hospital, Medical School of Nanjing University, No. 305 Zhongshan East Road, Nanjing, 210002 Jiangsu, China; ^2^Department of Intensive Care Unit, Suzhou Hospital Affiliated to Nanjing Medical University, No. 16 Baita West Road, Suzhou, 215002 Jiangsu, China; ^3^Hope Assist Medical Assistance Service Company, No. 1111 Tower A, Chaoyang SOHO, Beijing 100020, China; ^4^Department of Gastroenterology, Affiliated Hospital of Yangzhou University, No. 386 Hanjiang Media Road, Yangzhou, 225000 Jiangsu, China

## Abstract

**Objective:**

Severe acute pancreatitis (SAP) patients usually develop persistent organ dysfunction which causes the majority of deaths. It is important for SAP patients to receive centralized diagnosis and treatment in an experienced tertiary center. China, as a vast country with uneven distribution of medical resources, should take advantage of air medical transportation to meet the challenge of patient transfer among different regions. The aim of this study was to evaluate the safety and effectiveness of air transport for SAP patients via extra long distance.

**Methods:**

This was a retrospective analysis of all air medical transportations for SAP patients admitted to Jinling Hospital from January 2010 to December 2016. The general characteristics, transportation process, and clinical outcomes of these patients were recorded, and the safety and effectiveness of air transport were evaluated.

**Results:**

All the 20 SAP patients were successfully transferred by chartered aircraft without any occurrence of severe transport-associated complications. The mean transport time was 5.86 hours and the average transport distance was 1530 kilometers. The majority of SAP patients got timely intervention and the ultimate mortality rate was 15%.

**Conclusions:**

Air medical transport appears to be safe and effective for SAP patients with vital organ dysfunctions during the extra long-distance transportation.

## 1. Introduction

Acute pancreatitis (AP) is a severe inflammatory disorder of the pancreas and one of the most fatal diseases of the gastrointestinal tract around the world. Recent studies showed that the annual incidence of AP varied between 13 and 45 cases per 100,000 people [1]. The majority of AP patients have a mild disease course and recover within several days; however, about 15%–20% of the patients will develop severe AP (SAP) [2–4]. Persistent organ failure poses the greatest threat and causes the majority of mortality cases in SAP patients [5]. In view of the low incidence and the complicated pathophysiological process of SAP, the Japanese guideline recommends that severe cases should be transferred immediately to a facility capable of providing treatment for SAP [6].

The Jinling Hospital SAP treatment center was established in 1997. In recent years, more than 300 SAP patients have been admitted each year and till now, more than 2000 cases have been admitted to our center of whom the mortality rate was below 5%. Our center is located in eastern China, and the extra long distance (in this study defined as over 1000 kilometers) between our center and the remote areas has hindered these remote SAP patients to get timely and effective treatment measures. Hence, it is of great challenge to transfer these SAP patients to our center safely and quickly. In the past, patients were mainly transported by ambulances which had the disadvantages of long travel time, limited onboard equipment and bumpy ride. These problems restricted the application of ambulances in SAP patient transportation over an extra long distance [7]. It usually took ambulances more than a whole day to transfer patients to our center which was extremely dangerous for SAP patients, hence most of them had to give up transportation. A few transferred patients had worsened disease conditions because of the lack of monitoring of close vital signs and lack of organ function support during the distant transport process.

The aforementioned dilemma had stimulated the growing need for air medical transport. Air medical transport in China had been put into practice for decades and experienced accelerating demand in the past few years [8]. There is no doubt that air transfer has created a new era for the transportation of SAP patients. However, controversy has existed for a long time regarding the safety, benefits, and potential risks of air transportation [9, 10]. Although SAP patients often benefit from transfer to tertiary care for services not available at the sending hospital, the risks of adverse clinical outcomes associated with transport and aviation accidents cannot be ignored [11]. Hence, it is of great importance to assess the safety and explore the benefits and risks of air transport for SAP patients over an extra long distance, considering the lack of relevant literature reported until now.

In the present study, we aimed to analyze cases that underwent air medical transport between 2010 and 2016 in terms of general characteristics, transportation process, transport-related data, and clinical outcomes, as well as to assess the safety of air transfer for SAP patients over an extra long distance.

## 2. Materials and Methods

This was a retrospective analysis of all air medical transportations for SAP patients admitted to Jinling Hospital, Medical School of Nanjing University from January 2010 to December 2016. Diagnosis and disease severity classification of AP were according to the Revised Atlanta Classification [12]. Inclusion criteria were SAP patients with single or multiple persistent organ failure (>48 h) and with transport distances over 1000 kilometers. It was important to note that all transport of patients was carried out by professional medical transfer organizations, after a request from the next of kin or legal guardian of the patient. This study was approved by the institutional review board of Jinling Hospital. Our study was carried out in accordance with the relevant guidelines and regulations of our hospital and oral informed consent was obtained from all participants. The disease-associated data before and during the transport were recorded and provided by the professional medical staff from Hope Assist Medical Assistance Service Company, and disease-associated data after the transport were recorded in our treatment center.

### 2.1. Definition

#### 2.1.1. Systemic Complication

The definitions of organ dysfunction were based on a score of ≥2 in the sequential organ failure assessment (SOFA) scoring system. According to the Third International Consensus Definitions for Sepsis and Septic Shock (Sepsis-3), sepsis should be defined as a life-threatening organ dysfunction caused by a dysregulated host response to infection [13].

#### 2.1.2. Local Complications

Apart from the common complications (acute peripancreatic fluid collection, pancreatic pseudocycts, acute necrotic collection, and walled-off necrosis) which involve the pancreas itself, in this study, we focused more on severe AP-induced complications, including intra-abdominal hemorrhage, gastrointestinal fistula and portal vein thrombosis [12].

#### 2.1.3. Transfer Time and Transfer Distance

Transfer time was defined as time from actual delivery of the patient at the sending hospital to handing over to the primary medical team at our hospital. Transfer distance was defined as the sum of ground transportation distance from the sending hospital to the discharge disposition in the airport, intercity flight distance, and ground transportation distance from the admitting disposition in the airport to our hospital.

#### 2.1.4. Vital Signs of Transferred SAP Patients before and after Transport

Vital signs of transferred SAP patients before and after transport (Table 1) refer to the vital signs and blood gas analysis recorded before departing from the sending hospital and the vital signs and blood gas analysis recorded immediately after arriving at our hospital.

#### 2.1.5. The Definition of Abnormal Events of Vital Signs during Transfer (Table 2)

The baseline values of vital signs refer to the values recorded for the first time during the transfer process. The heart rate, respiratory rate, blood pressure, and blood oxygen saturation were automatically measured by the ECG monitor, the axillary temperature was measured by the thermometer, and the urine volume per hour was collected by the sterile catheter and recorded by the nurse.

#### 2.1.6. Increased Body Temperature

Axillary temperature was measured every half hour and if the temperature of a single measurement increased by 0.5 degrees compared to the baseline value, it was defined as an increased in body temperature.

#### 2.1.7. Heart Rate Elevation

If the heart rate was elevated by 20% compared to the baseline value and lasted longer than 10 min, it was defined as heart rate elevation.

#### 2.1.8. Tachypnea

If the respiratory rate was elevated by 5 times per minute compared to the baseline value and lasted longer than 10 min, it was defined as tachypnea.

#### 2.1.9. Decreased Blood Pressure

Noninvasive blood pressure was measured every 10 minutes, and if the average arterial pressure dropped below 65 mmHg for two consecutive measurements, it was defined as decreased blood pressure.

#### 2.1.10. Decreased Blood Oxygen Saturation

If oxygen saturation dropped by 5% compared to the baseline value and lasted longer than 5 minutes, it was defined as decreased blood oxygen saturation.

#### 2.1.11. Decreased Urine Volume

If the urine volume was decreased by 10 ml/h compared to the baseline value for two consecutive measurements, it was defined as decreased urine volume (except for AKI patients with anuria).

### 2.2. Aircraft

**Figure 1:**
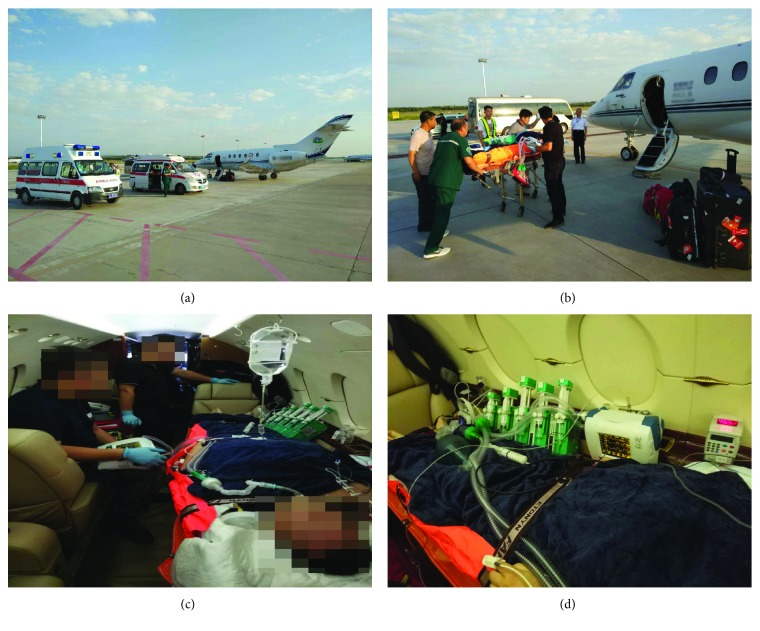
The emergency air medical transport of severe acute pancreatitis patients.

**Figure 2:**
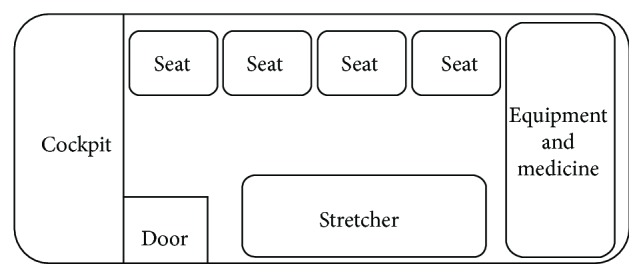
The general customized arrangement of an eight-seat chartered aircraft for medical transport. It can accommodate one doctor, one nurse, one accompanying person, and the patient on a stretcher.

Small fixed-wing and rotary aircraft (see Figure 1) available for the transfer of patients included the following types: HAWKER800, Alouette III; L-3; Astar; Twin Star; BO-105; AgustaWestland A109; BK-117; Bell 222; Dolphin; and S-76. Most of them can accommodate one doctor, one nurse, one accompanying person, and the patient on a stretcher (see Figure 2).

### 2.3. Equipment

Standard equipment for air medical transport includes transfer facilities (stretcher, air cushion, cervical collar, fracture-fixing apparatus, etc.), medical monitoring instruments (electrocardiogram monitor, pulse oximeter, etc.), airway management devices (oxygen supply equipment, medical ventilator, electric-drive phlegm-sucker, tracheal intubation kit, endotracheal tube, oropharyngeal airway, tracheotomy kit, atomization inhalation kit, etc.), vascular access devices (intravenous needle, bagged intravenous infusion, etc.), surgical treatment devices (bandage, dressing, gloves, masks, minor surgery kit, simple disinfection kit, etc.) and pregnancy- and delivery-associated devices (fetal Doppler monitor, delivery kit, neonatal rescue kit, etc.). An intra-aortic balloon pump (IABP), cardiac pacing device, and cardiac electric defibrillator are also prepared according to specific circumstances. A satellite phone is used to communicate with outside world while on the aircraft. All equipment is guaranteed to be compact, portable, and noninterfering with radio communications according to aviation standards.

### 2.4. Medicine in Storage

Standard medications for air medical transport include ATP-adenosine 20 mg/2 ml; adenosine 6 mg/2 ml; amiodarone 150 mg/3 ml; atropine 5 mg/10 ml; metoprolol 5 mg/5 ml; diltiazem 10 mg; dexamethasone 5 mg/1 ml; diazepam 10 mg/2 ml; diphenhydramine 50 mg/1 ml; dopamine 200 mg/5 ml; furosemide 20 mg/2 ml; 50% glucose 20 ml; heparin 1000 units/1 ml; hyoscine 20 mg/ml; KCL 1.5 g/10 ml; labetalol 25 mg/5 ml; lignocaine 100 mg/5 ml; metoclopramide 10 mg/2 ml; nitroglycerin 10 mg/10 ml; naloxone HCL 0.4 mg; normal saline 30 ml for injection; omeprazole 40 mg; paracetamol 250 mg/suppository; phenobarbital, 65 mg/ml or 130 mg/ml; salbutamol 0.5 mg/ml; sterile water, 30 ml for injection; tramadol 50 mg; terbutaline, 1 mg/1 ml; and verapamil, 5 mg/2 ml.

### 2.5. Procedure of Air Medical Transport

**Figure 3:**
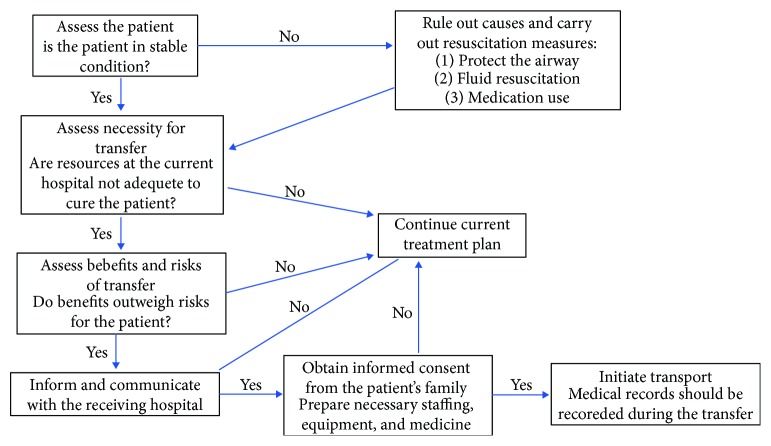
The flowchart to guide the transfer process of severe acute pancreatitis patients.

According to the flowchart developed by us to guide the transfer process of SAP patients (Figure 3), firstly, it is important to assess the patient condition and if the patient is in an unstable condition, necessary resuscitation measures should be provided. Then, the necessity for interhospital transfer needs to be assessed. When the resources at the current hospital are inadequate to address the patient's needs and the benefits of the transfer outweigh the risks, the patient had better be transferred to more capable hospitals. Afterwards, responsible doctors at the current hospital need to contact the receiving hospital and obtain informed consent from the family. What's more, inclusion into the flight line should be applied for in advance and approval from the China Air Force command should be obtained. If all goes according to plan, the transport process could be initiated as soon as possible and necessary medical records should be maintained among the transfer process.

### 2.6. Statistical Analysis

The SPSS 22.0 statistical software package (IBM Analytics, Armonk, NY) was used for statistical analyses. Data are presented as mean ± standard deviation (SD) for continuous variables and absolute numbers and percentages for categorical variables. Student's *t*-test or Mann–Whitney test was used for analyzing continuous variables and the Chi-square test was used for analyzing categorical variables.

## 3. Results

From January 2010 to December 2016, a total of 29 patients with SAP were transferred by air medical transportation to our SAP treatment center. They included 6 patients who were excluded for the utilization of air transport vehicles other than chartered aircraft (4 patients transferred by the stretchers in the commercial flights, 2 patients transferred by helicopters), 1 patient who was excluded because the transport distance was less than 1000 kilometers, and 2 other patients who were excluded due to the disease severity which was moderately severe AP. Finally, 20 SAP patients were included in our study. Their baseline characteristics and clinical features are shown in Table 3. The mean age of the patients was 42 years (range 23–72 years). 6 (30%) of these were women and 14 (70%) were men. The etiologies of AP are as follows: gallstone in 8 (40%) patients, hypertriglyceridemia in 10 (50%) patients, alcohol abuse in 1 (5%) patient, and trauma in 1 (5%) patient. The average body mass index was 26.8 (range 21.3–36.1), the average Acute Physiology and Chronic Health Evaluation II score was 17.1 (range 6–27), the average Sequential Organ Failure Assessment score was 6.2 (range 2–9), and the average Balthazar CT score was 8.5 (range 4–10). The mean time interval from pancreatitis onset to transportation was 27.6 days (range 8–66 days). All 20 (100%) patients were defined as severe AP on the basis of the severity of AP according to the Revised Atlanta Classification. All the 20 patients developed local or systemic complications before transportation. The most common systemic complications were acute respiratory distress syndrome (ARDS) and acute kidney injury (AKI). 16 (80%) patients suffered from ARDS and received mechanical ventilation, 18 (90%) patients developed AKI and needed renal replacement treatment, and 12 (60%) patients suffered from shock and were administrated vasoactive drugs. According to the updated Sepsis 3.0 definition, sepsis is defined as an infection plus a SOFA score ≥ 2; hence, the occurrence rate of sepsis in the 20 patients was 100%. As for severe AP-associated local complications, infected pancreatic necrosis was observed in all 20 (100%) patients, and intra-abdominal hemorrhage was observed in 6 (30%) patients. 2 (10%) patients were diagnosed with portal thrombosis and 3 (15%) patients developed gastrointestinal fistula during their disease course before transport. In terms of the treatment for infected pancreatic necrosis, 13 (65%) patients underwent invasive drainage, and 8 (40%) patients were performed with surgical operations. Sedative agents and analgesics were used in the majority of the transferred patients.

Transport-associated data and abnormal conditions that occurred during the transfer were recorded in Table 2. The average time spent for transport was 5.86 hours (range 3.5–8 hours) and the average transport distance was 1530 kilometers (range 1000–2300 kilometers). During the transport process, important medical records, for example, vital signs were monitored closely. Alterations in vital signs that occurred in the transfer process included the following: tachycardia, which happened in 3 (15%) patients and were handled with antiarrhythmic drugs discreetly; decreased blood pressure, which happened in 2 (10%) patients and vasoactive agents were applied to maintain steady blood pressure; fever, which happened in 4 (20%) patients and physical or drug cooling was used; SPO_2_ reduction, which happened in 1 (5%) patient and was handled by adjusting the parameters of the mechanical ventilation; and 2 (10%) patients which showed decreased urine volume and were carefully observed.

Vital signs of transferred SAP patients before and after transport were recorded and compared in Table 1. It showed no significant alterations in vital signs between the two groups.

In terms of clinical course and outcomes (Table 4), the overall mortality rate was 15% in the 20 transferred SAP patients. The mean length of ICU stay and hospital stay were 46 days (range 8–116 days) and 65 days (range 15–175 days), respectively. During the hospitalization in our center, 20 (100%) patients developed MODS eventually and 17 (85%) patients received mechanical ventilation, 18 (90%) received renal replacement therapy, and 18 (90%) patients were managed with inotropic agents. In the management of infected pancreatic necrosis [14], 17 (85%) patients underwent percutaneous catheter drainage, then 8 (40%) patients underwent double-tube continuous negative-pressure suction, 3 (15%) patients underwent endoscopic necrosectomy, and 12 (60%) patients underwent open necrosectomy.

## 4. Discussion

The treatment of SAP patients is complicated and often requires multidisciplinary collaboration, which involves a gastroenterologist, general surgeon, ICU doctor, and an interventional radiologist. Furthermore, as to the SAP patients with organ dysfunction or infected pancreatic necrosis, it is vital to handle with organ function and conduct pancreatic necrotic tissue drainage or necrosectomy, which may not be that easy to operate for doctors from a single department [15, 16]. Hence, it is of great importance for SAP patients to be transferred to a professional and experienced treatment center to receive specialized care.

The decision to transport a critically ill patient to another medical facility is based on the careful assessment of the potential benefits of transport weighed against the risks [17, 18]. In this study, SAP patients were transported to our treatment center to obtain additional care which was not available at the sending hospital. All the 20 patients were diagnosed as IPN combined with a single or multiple persistent organ failure, as well as the presence of other severe complications, including hemorrhage and gastrointestinal fistula. After the timely and effective treatment in our center, the final in-hospital mortality rate of these patients was 15%. The transport most likely altered the management and the outcomes of the SAP patients, and there was no doubt that the majority of the patients obtained survival benefits from the transport.

This was the first report to show that not only was there no air crash in 20 flights for SAP patients' transportation in six years over an extra long distance (≥1000 kilometers), but there was also no fatal transport-associated complications occurring during the transport. This demonstrated the effectiveness of patient safety and flight safety with a professional air medical transfer team. The transport of critical patients by air has become an integral part of healthcare systems [19]. Previous studies have stated that the development of effective time-critical interventions for acute disease patients, especially acute myocardial infarction and ischemic stroke, had improved the outcomes [20]. In our study, given the transport distance and the need for timely intervention, SAP patients with critical organ dysfunctions would undoubtedly benefit from the transport. There are several merits that ensure the safety and effectiveness of air transport: (1) the short time spent for the extra long-distance transport, (2) a chartered aircraft which has a relatively stable flight process compared to helicopters, (3) a mobile ICU with advanced medical equipment [21], (4) a professional transport team with experienced and high-quality personnel, and (5) a systemic and normative transport flowchart.

Critically ill patients are at increased risk of high morbidity and mortality during air transport. However, risks could be minimized and outcomes improved with careful planning, appropriate allocation of qualified personnel, and appropriate use of equipment and application of medication. During transport, it is of first priority to monitor and maintain the stability of patients' vital functions, for example, to ensure the effective ventilation of the airway and to keep blood pressure stable. Furthermore, the accompanying doctors and nurses are trained to handle emergency conditions and provide acute care needs for the patients [22].

The major risks of air transporting SAP patients are the low atmospheric pressure and gas expansion effect of flying altitude as well as the long-distance movement-related complications [23]. With the increase of flight height, the atmospheric pressure changes extremely and in the low-pressure environment, the gas stored in the gastrointestinal tract expands and causes gastrointestinal flatulence. This adverse consequence really becomes an added insult to SAP patients, which exacerbates the condition of their preexisting intra-abdominal hypertension. What make the things worse is the increase in intra-abdominal pressure that leads to limited breathing and even dyspnea, and into a vicious cycle in ARDS patients. In addition, the decrease in PiO_2_ seems to be not easily tolerated by critically ill patients with limited reserves, which causes hyperventilation and tachycardia with an increase in cardiac output [24]. Hence, these adverse effects remind us to monitor and maintain the respiratory problem and intra-abdominal hypertension of SAP patients discreetly. Luckily, in our study, during the air transportation of SAP patients, none of them developed fatal transport-associated respiratory and cardiovascular complications; however, in a proportion of the patients, tachycardia, fluctuation of blood pressure, and SPO_2_ reduction occurred which were handled discreetly.

Over the past decades, China has witnessed an explosive increase in interfacility air medical transport. However, despite that air transport is more likely to show its superiority in extra long-distance transfer, it has these limitations: (1) There must be airports near the hospitals involved in the transport process. (2) Inclusion into the flight line should be applied in advance. (3) It is money consuming and not covered by medical insurance in China. Despite the efficacy and safety of air medical transport, it is hard to satisfy the needs of the majority of SAP patients, hence, as for these extra long-distance transport patients, more transfer modes should be developed to complement the medical transport system.

In recent years, railway construction has been extremely rapid and complete, and China really ushered in the age of the high-speed railway system [25]. In view of the time spent for getting stuck in the airport and the super high speed of the modern railway, high-speed railway medical transport seems to be more stable, timesaving, and money saving than air transport. High-speed railway transport shows the following superior qualities of long-distance transport (>500 km): (1) Railway stations cover the majority of cities in China and is much more widespread than airports. (2) There are many trains going back and forth between two cities and it really saves a lot of time without complicated screening procedures. (3) The speed of a railway averages up to 300 km/h and it is timesaving. (4) The cost of the railway transport is extremely lower than airplanes, and is within the economical reach of most Chinese people. Hence, we have reasons to believe that the high-speed railway will be used for medical transport in the future and will undoubtedly play an important role in critically ill patients' transportation.

### 4.1. Limitations

Limitations of this study include the small number of SAP cases via air transport, a lack of a control group of AP patients transported by ground ambulance, as well as a lack of records and analyses of weather and traffic conditions at the time of each transfer.

In conclusion, during the extra long-distance transportation for SAP patients with vital organ dysfunctions, air medical transport appears to be an alternative choice to ensure that patients receive timely intervention and obtain better clinical outcomes.

## Figures and Tables

**Table 1:** Vital signs of transferred severe acute pancreatitis patients before and after transport.

Variables, number (%)	Before transfer	After transfer	*P* value
Heart rate			0.749
≤120 beats/min	12 (60)	11 (55)	
>120 beats/min	8 (40)	9 (45)	
Blood pressure			0.788
Without vasopressors	8 (40)	7 (35)	
Norepinephrine ≤ 0.1 *μ*g/kg/min	7 (35)	6 (30)	
Norepinephrine > 0.1 *μ*g/kg/min	5 (25)	7 (35)	
PaO_2_/FiO_2_ (mmHg)			0.746
>200	4 (20)	4 (20)	
100–200 and mechanically ventilated	12 (60)	10 (50)	
<100 and mechanically ventilated	4 (20)	6 (30)	
Urine volume			1
Anuria	12 (60)	12 (60)	
≤40 ml/h	6 (30)	6 (30)	
>40 ml/h	2 (10)	2 (10)	
pH			0.709
>7.45	4 (20)	3 (15)	
7.35–7.45	13 (65)	12 (60)	
<7.35	3 (15)	5 (25)	
Lactate			0.744
≤2	13 (65)	12 (60)	
>2	7 (35)	8 (40)	
PaCO_2_			0.926
>45	4 (20)	5 (25)	
35–45	12 (60)	11 (55)	
<35	4 (20)	4 (20)	

Categorical variables were presented as absolute numbers and percentages.

**Table 2:** Transport-associated data of the severe acute pancreatitis patients transferred to our center.

Variables	All SAP patients
Transport time (hours)	5.86 (1.19)
Transport distance (kilometers)	1530 (433)
Alterations in vital signs	
Increased body temperature	4 (20)
Heart rate elevation	3 (15)
Decreased blood pressure	2 (10)
Tachypnea	4 (20)
Decreased blood oxygen saturation	1 (5)
Decreased urine volume	2 (10)

Data are presented as mean ± standard deviation for continuous variables and absolute numbers and percentages for categorical variables.

**Table 3:** Baseline characteristics and clinical features of the severe acute pancreatitis patients before transferred to our center.

Variables	All AP patients
Age (years)	42 (14)
Gender (male/female)	14/6
BMI	26.8 (4.11)
APACHE II score	17.1 (6.22)
SOFA score	6.2 (1.98)
CT severity index	8.5 (1.90)
Disease course when transfered (days)	27.6 (16.5)
Etiology of acute pancreatitis, number (%)	
Gallstone	8 (40)
Hypertriglyceridemia	10 (50)
Alcohol	1 (5)
Trauma	1 (5)
Other	0 (0)
Severity of AP, number (%)	
Mild	0 (0)
Moderately severe	0 (0)
Severe	20 (100)
Systemic complication, number (%)	
ARDS	16 (80)
AKI	18 (90)
Shock	12 (60)
Sepsis	20 (100)
Local complication, number (%)	
IPN	20 (100)
Intra-abdominal hemorrhage	6 (30)
Portal thrombosis	2 (10)
Gastrointestinal fistula	3 (15)
Treatment, number (%)	
Mechanical ventilation	16 (80)
CRRT	18 (90)
Vasoactive drugs	14 (70)
Sedative treatment	14 (70)
Analgesics	12 (60)
Invasive drainage	13 (65)
Operation	8 (40)

BMI—body mass index, APACHE II—acute physiology and chronic health enquiry II, SOFA—sequential organ failure assessment, CT—computed tomography, ARDS—acute respiratory distress syndrome, AKI—acute kidney injury, IPN—infected pancreatic necrosis, CRRT—continuous renal replacement therapy. Data are presented as mean ± standard deviation for continuous variables and absolute numbers and percentages for categorical variables.

**Table 4:** Clinical course and outcomes of severe acute pancreatitis patients transferred to our center.

Variables	All AP patients
IPN treatment	
PCD, number (%)	17 (85)
DCNP, number (%)	8 (40)
Endoscopic necrosectomy, number (%)	3 (15)
Open necrosectomy, number (%)	12 (60)
MODS treatment	
Mechanical ventilation	17 (85%)
Renal replacement therapy	18 (90%)
Inotropic agents	18 (90%)
Mortality, number (%)	3 (15)
Hospital duration (days)	65 (41.3)
ICU duration (days)	46 (30.6)
Cost (thousand CNY)	476 (267)

ICU—intensive care unit, IPN—infected pancreatic necrosis, PCD—percutaneous catheter drainage, DCNP—double-tube continuous negative pressure suction, MODS—multiple organ dysfunction syndrome. Data are presented as mean ± standard deviation for continuous variables and absolute numbers and percentages for categorical variables.

## Data Availability

The data in this study are available for other researchers to verify the results of our article, replicate the analysis, and conduct secondary analyses. Other researchers can send e-mails (e-mail addresses: njzyantol@hotmail.com or lwqsaplab@163.com) to contact us to obtain our data.

## Abbreviations

SAP: Severe acute pancreatitis
SOFA: Sequential organ failure assessment
IPN: Infected pancreatic necrosis
AKI: Acute kidney injury
ARDS: Acute respiratory distress syndrome
MODS: Multiple organ dysfunction syndrome
ICU: Intensive care unit.
